# Assessment of a Computational Approach to Predict Drug Resistance Mutations for HIV, HBV and SARS-CoV-2

**DOI:** 10.3390/molecules27175413

**Published:** 2022-08-24

**Authors:** Dharmeshkumar Patel, Suzane K. Ono, Leda Bassit, Kiran Verma, Franck Amblard, Raymond F. Schinazi

**Affiliations:** 1 Center for ViroScience and Cure, Laboratory of Biochemical Pharmacology, Department of Pediatrics, Emory University School of Medicine and Children’s Healthcare of Atlanta, 1760 Haygood Dr., Atlanta, GA 30322, USA; 2 Department of Gastroenterology, University of São Paulo School of Medicine, Av. Dr. Arnaldo, 455, São Paulo 05403-000, SP, Brazil

**Keywords:** HIV, HBV, SARS-CoV-2, 3CLpro, RT, capsid, drug resistance, mutation, residue scanning, MM-GBSA, emtricitabine, nirmatrelvir

## Abstract

Viral resistance is a worldwide problem mitigating the effectiveness of antiviral drugs. Mutations in the drug-targeting proteins are the primary mechanism for the emergence of drug resistance. It is essential to identify the drug resistance mutations to elucidate the mechanism of resistance and to suggest promising treatment strategies to counter the drug resistance. However, experimental identification of drug resistance mutations is challenging, laborious and time-consuming. Hence, effective and time-saving computational structure-based approaches for predicting drug resistance mutations are essential and are of high interest in drug discovery research. However, these approaches are dependent on accurate estimation of binding free energies which indirectly correlate to the computational cost. Towards this goal, we developed a computational workflow to predict drug resistance mutations for any viral proteins where the structure is known. This approach can qualitatively predict the change in binding free energies due to mutations through residue scanning and Prime MM-GBSA calculations. To test the approach, we predicted resistance mutations in HIV-RT selected by (-)-FTC and demonstrated accurate identification of the clinical mutations. Furthermore, we predicted resistance mutations in HBV core protein for GLP-26 and in SARS-CoV-2 3CLpro for nirmatrelvir. Mutagenesis experiments were performed on two predicted resistance and three predicted sensitivity mutations in HBV core protein for GLP-26, corroborating the accuracy of the predictions.

## 1. Introduction

The discovery of effective antiviral drugs revolutionized world health by delaying virus-associated disease progression and thus saving millions of lives. Despite these medical advances, the selection of drug-resistant strains is a persistent problem that leads to viral breakthrough and reduced antiviral efficacy [1,2,3,4]. There are several mechanisms reported for the development of drug resistance [5,6]. Random mutations in viral genes which alter the binding of the drug to its protein target are the primary mechanism of acquired drug resistance in viruses [6]. The mutation rate in RNA viruses is generally very high—estimated at 10^−4^ per nucleotide per replication. In contrast, DNA viruses have an estimated mutation rate of 10^−8^ per nucleotide per replication [7,8]. The primary cause of failure of anti-HIV therapy is the selection of drug-resistant mutants. With the advent of genetic sequencing and a deeper understanding of drug resistance mechanisms, combination drug therapy has become the standard of care [9,10]. Similarly, the use of multiclass combination therapy in HCV effectively prevented the selection of resistant mutants, leading to curative rates in the range of 98% [11]. Thus, the emergence of drug-resistant viruses is one of the greatest risks to public health and is a priority across the globe. Foreknowledge of potential mutations that could be selected in vitro and in vivo, coupled with a comprehensive understanding of viral resistance mechanisms, is of immense importance in developing more effective and durable drug treatments.

Experimentally, drug-resistant viruses are typically selected by maintaining an infected culture under drug pressure for months (sometimes years) without a guarantee that resistance will emerge under those specific cellular conditions [12]. In certain situations, resistance appears exclusively in clinical settings, requiring hasty characterization of the mutation and viral species [13]. The ability to predict drug resistance mutations expedites understanding of antiviral efficacy, anticipates activity against existing mutant strains, delivers mechanistic insight into how specific mutants confer resistance, allows for the design of drug combinations that are not cross-resistant, forecasts mutant species that may develop in clinical settings, provides guidance on the development of diagnostic assays that detect mutations and generally provides broad utility and benefit to infectious disease drug discovery [14].

Numerous efforts have been made to develop tools to predict drug resistance mutations. One group of prediction models includes sequence-based approaches, which use various machine learning methods. These prediction models rely primarily on primary sequences of the protein or genotypic sequence data, and their prediction accuracies are dependent on the availability of large and diverse training sets [15,16,17,18]. The main advantage of these methods is that they are computationally efficient. A weakness of these methods is that they are reliant on the availability of training set data. Further, without 3-D structural information and knowledge of the enzymatic function of the mutated residues, this group of models fails to link viral genetic mutations and structural changes due to corresponding phenotypic mutations [14,19,20]. A second type of prediction models is based on the 3-D structure of the target proteins. In the last few decades, the availability of a large number of 3-D structures of protein targets has enabled the implementation of various structure-based molecular modeling approaches to study binding interactions and binding free energies of drug molecules with their corresponding protein targets. The binding free energies are crucial for facilitating the prediction of drug resistance mutations [21,22,23,24]. Although these methods are advantageous over sequence-based methods, they can be time-consuming or show low predictive accuracy. Hence, there is a need for novel structure-based methods with an optimum balance between computational efficiency and accuracy [25].

Molecular mechanics–generalized Born surface area (MM-GBSA) is one of the structure-based approaches widely used to estimate binding affinities in protein–ligand or protein–protein complexes [26,27,28,29]. Recently, Schrödinger utilized a physics-based scoring function together with the MM-GBSA model (Prime MM-GBSA) to calculate changes in the binding free energy of protein–protein complexes due to single point mutations, which was called residue scanning [26,27]. Moreover, it was also shown that Prime MM-GBSA has slightly better accuracy compared to other prediction methods such as PoPMuSiC^syn^ [30], FoldX [31] and Rossetta [32] in predicting binding affinities due to single point mutations in protein–protein complexes [26,27]. Although promising, the ability of Prime MM-GBSA to calculate binding affinity due to single amino acid mutations and to predict resistance mutations based on interactions between small molecules and their protein targets has not been explored thoroughly.

Typically, drug-selected resistance mutations in viruses meet three requirements: (1) a decrease in the inhibitor binding affinity, (2) retention of the native substrate binding affinity to maintain essential viral function and (3) accessibility via a single nucleotide substitution (SNS) in the wild-type codon [19,33,34]. Very few active-site mutations meet all three criteria, providing the potential to efficiently predict resistance mutations. Moreover, these criteria can be analyzed computationally using structure-based methods. Herein, we describe the prediction of the binding affinities with the help of two Schrödinger suite modules, Residue Scanning and Prime MM-GBSA, in the context of predicting mutations conferring resistance to small molecule drugs following single amino acid mutations. We first implemented and validated this approach by predicting known resistance mutations for the approved HIV reverse transcriptase (RT) inhibitor (-)-FTC. Second, we used the approach to predict resistance mutations associated with GLP-26, a known HBV capsid assembly modulator (CAM)-26, and with nirmatrelvir, an emergency FDA-approved SARS-CoV-2 3CL protease inhibitor. Five GLP-26 resistance and sensitivity mutations were validated in mutagenesis experiments.

## 2. Results and Discussion

**Figure 1:**
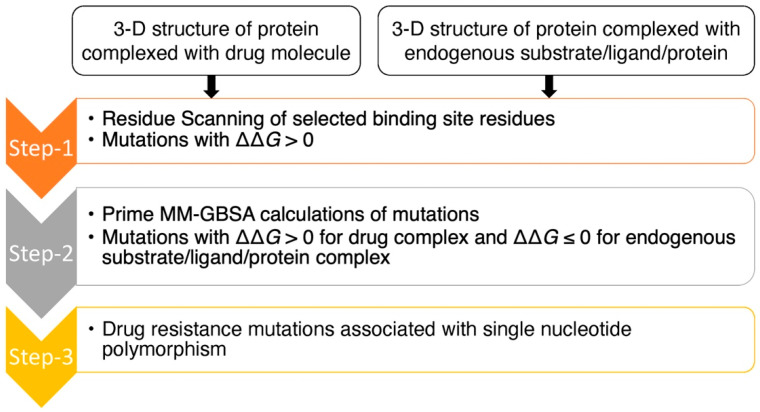
Flow chart of computational approach for the prediction of drug resistance mutations.

The purpose of this study was to assess the prediction accuracy of drug resistance mutation identification using the computational workflow shown in Figure 1. This workflow utilizes both residue scanning and Prime MM-GBSA calculations with the goal of optimizing the balance between predictive accuracy and computational efficiency. This approach was used on three protein–drug complexes: (1) HIV RT complexed with (-)-FTC-TP, (2) HBV core protein complexed with GLP-26 and (3) SARS-CoV-2 3CLpro complexed with the protease inhibitor nirmatrelvir.

The approach began with residue scanning followed by Prime MM-GBSA calculations (Figure 1). Residue scanning generated mutations for specified residues using the Prime rotamer search algorithm. We then performed Prime MM-GBSA refinement of the bound and unbound state for each system for both wild-type and mutant protein structures. We kept the protein backbone and the neighboring side chains fixed, allowing for rapid screening of the mutations and predicted binding affinities in a computationally efficient way. The main goal of residue scanning was to filter out mutations with increased drug/substrate binding affinities (ΔΔ*G* < 0 kcal/mol) early on and to keep only mutations with a decrease in binding affinities (ΔΔ*G* > 0 kcal/mol), allowing binding affinities with side-chain flexibility in the binding sites to be explored using Prime MM-GBSA at a later time point. Mutations with increasing binding affinities (ΔΔ*G* < 0 kcal/mol) are in energy minimum conformations [26], and we hypothesized that incorporating side-chain flexibility would be less likely to decrease binding affinities, and to therefore have less probability of changing binding free energies from a negative value (ΔΔ*G* < 0 kcal/mol) to a positive value (ΔΔ*G* > 0 kcal/mol). Moreover, mutations with ΔΔ*G* > 0 kcal/mol for the drug complexes and ΔΔ*G* ≤ 0 kcal/mol for the native substrate complexes (i.e., decreasing binding affinities for the drug molecules while maintaining or increasing binding affinities for substrates) are targeted as they could be potential resistance mutations.

In the next step, Prime MM-GBSA calculations were implemented with side-chain flexibility to calculate drug/substrate binding affinities with their point-mutated protein targets. Point-mutated structures, in which the mutations have ΔΔ*G* > 0 kcal/mol for drugs/substrates, were identified from the residue scanning step. Side-chain flexibility was provided for the residues located within 8 Å of the drugs/substrates to explore the conformational space of the side chains within the binding sites due to point mutations. Moreover, binding affinities for mutations with ΔΔ*G* > 0 kcal/mol could be improved from ΔΔ*G* > 0 kcal/mol to ΔΔ*G* ≤ 0 kcal/mol by incorporating side-chain flexibility in the binding sites. With Prime MM-GBSA, the binding affinity (Δ*G*) of a drug/substrate with wild-type and mutant protein targets is calculated separately to determine a free energy change (ΔΔ*G*). Mutations that maintain/increase binding affinities of the substrates (ΔΔ*G* ≤ 0 kcal/mol) and decrease binding affinities of the drug molecules (ΔΔ*G* > 0 kcal/mol) are potential drug resistance mutations. Most antiviral drugs are known to have low genetic barriers, which means that viruses can become resistant [34] through non-synonymous single-nucleotide polymorphism (SNP). Therefore, amino acid mutations associated with single-nucleotide polymorphisms were prioritized as possible drug resistance mutations.

**Figure 2:**
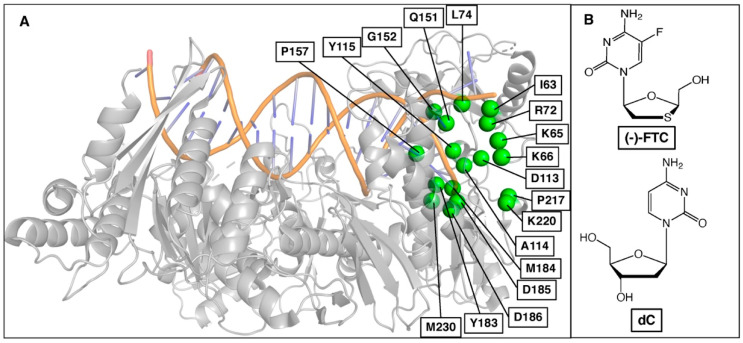
(**A**) Selected binding site residues in HIV-RT to predict resistance mutations for (-)-FTC. (**B**) Chemical structures of (-)-FTC and natural substrate 2′-deoxycytidine (dC).

To evaluate our approach, we used (-)-FTC (emtricitabine), which in its 5′-triphophate form ((-)-FTC-TP) is a well-characterized HIV reverse transcriptase (HIV-RT) inhibitor, to determine if we were able to recapitulate its mutation profile. HIV-RT polymerizes the viral DNA primer from an RNA template. To do so, the active site binds 2′-deoxynucleotide triphosphates, such as 2′-deoxcytidine triphosphate dCTP (Figure 2), for chemical incorporation into the growing DNA strand [35]. Nucleoside analogs have been developed that bind to HIV-RT and terminate DNA chain elongation after incorporation. (-)-FTC is a frontline nucleoside analog in antiretroviral therapy [36,37,38]. The drug is converted to the active nucleoside triphosphate form by host kinases, and the active nucleoside triphosphate form then outcompetes dCTP for binding to HIV-RT and terminates genome chain polymerization. The pharmacological activities and resistance mutations of (-)-FTC were first described and studied rigorously by Schinazi et al. [39,40]. Moreover, the clinically significant resistance mutations are reported and well-studied [41]. It is well established that (-)-FTC selects the M184V resistance mutation in the HIV-RT active site leading to virologic breakthrough [39,40]. Thus, HIV RT with (-)-FTC was the ideal system for testing the ability of our computational protocol to predict the resistance mutations.

**Figure 3:**
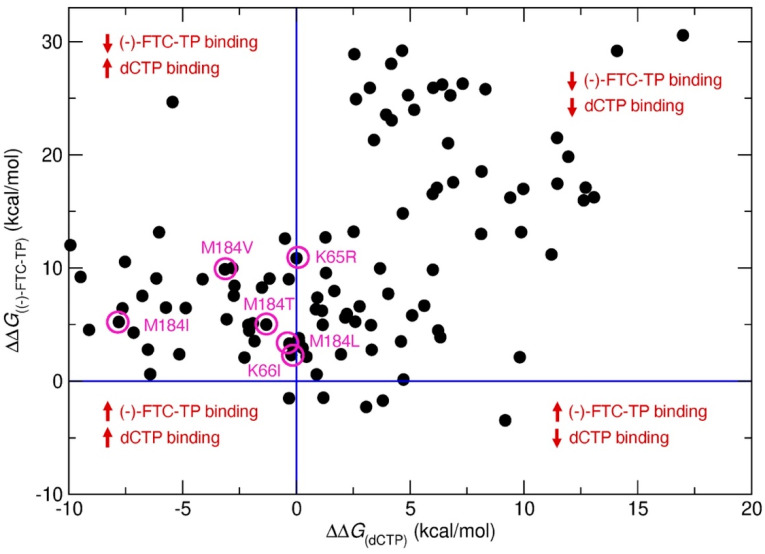
Predicted binding free energy change (ΔΔ*G*) in kcal/mol of native substrate dCTP versus (-)-FTC-TP for single point mutations in HIV-RT. Violet circles represent known clinical (-)-FTC resistance mutations.

The approach predicted 157 resistance mutations through the first step of residue scanning and 48 resistance mutations through the second step of Prime MM-GBSA calculations. This demonstrates that incorporation of side-chain flexibility in Prime MM-GBSA filtered out mutations that do not reduce drug/substrate binding affinities and that resistance mutations were selected. Finally, SNP mutations were selected as probable resistance mutations (Figure 3, Table 1). Figure 3 shows the predicted binding free energy changes of natural substrate dCTP (ΔΔ*G*_(dCTP)_) versus drug (-)-FTC-TP (ΔΔ*G*_(FTC-TP)_) obtained from Step 2. Mutations leading to a decrease in binding affinity of (-)-FTC-TP while maintaining or increasing the binding affinity of substrate dCTP are shown on the top left side of the graph. Thus, mutations in this region could be potential drug resistance mutations. This approach allowed us to recapture several clinically relevant resistance mutations, including M184V and M184I (Figure 3, Table 1), two mutations known to show 500–1000-fold resistance to (-)-FTC. Although the predicted ΔΔ*G* values do not correlate with experimental values, the predicted ΔΔ*G* values for M184V and M184I are higher relative to other mutations. Between M184V and M184I, the ΔΔ*G* value of M184V is higher than that of M184I, which corroborates the experimental data.

Moreover, four other clinically reported mutations, M184L, M184T, K65R and K66I, were also predicted using our approach, validating the accuracy of our method. However, it is worth noting that our model did not predict the Q151M mutation, a clinically known mutation to reduce 2-fold EC_50_ of (-)-FTC. Enzymatically, the Q151M mutation remains sensitive to (-)-FTC with the same activity [42], and our approach is based on target protein structure, providing a possible explanation for why this mutation was not identified through our approach. Although Q151M was not predicted, other Q151 mutations Q151E/I/C/D/P were predicted as resistance mutations for (-)-FTC. Other predicted resistance mutations from Table 1 have not yet been reported, but they could be put on a potential watchlist for resistance to (-)-FTC. The prioritized list of resistance mutations for (-)-FTC based on ΔΔ*G* values is summarized in supplementary materials.

**Figure 4:**
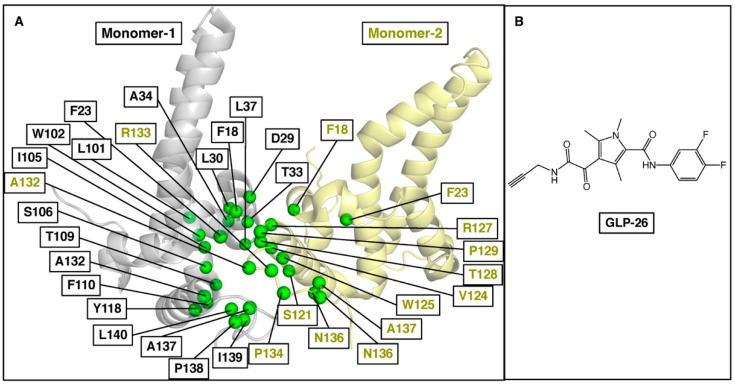
(**A**) Selected binding site residues between two monomer proteins of the HBV core to predict GLP-26 resistance mutations. The monomers are represented in gray and yellow, and their respective residues are in green. (**B**) Chemical structure of GLP-26.

To explore our predictive approach further, we turned our attention to the HBV capsid, which plays a pivotal role in the replication cycle of the virus. Capsid assembly modulators (CAMs) have been shown to bind between two monomeric core proteins and impair capsid assembly by affecting the kinetics and/or the binding strength between core protein dimers [43]. GLP-26 is a non-toxic, highly potent HBV CAM which displays promising effects in vitro and in various animal models [44,45,46]. Based on the unique profile of this compound, we decided to use our approach to predict its resistance mutation profile. As there is no substrate involved in the HBV capsid assembly, ΔΔ*G* values of two monomeric core proteins due to mutations were compared with and without GLP-26 complexes. The selected binding site residues from both monomers are shown in Figure 4.

Using our three-step workflow (Figure 1), we predicted a series of resistance mutations in HBV core protein for GLP-26 (Table 2). Interestingly, the F110I, T128I and L140I mutations have been reported for other CAMs [47], F101I (JNJ-6379 and Bay41-4109), T128I and L140I (JNJ-6379). These mutations were predicted to reduce GLP-26 binding affinity to a higher degree (ΔΔ*G* > 3 kcal/mol). Other known CAM-associated mutations including F23Y, T33Q, L37Q, I105T, I105V, Y118F, V124A and V124G have also been reported [47], but are predicted to show only mild to moderate effect on the binding of GLP-26. Finally, T109 mutations, known to be resistant to most HBV CAMs [47,48], are not predicted to be an issue with GLP-26 (Figure 4, Table 2) and could, therefore, provide options for combination therapies with other CAMs. To validate our prediction model and its accuracy, we performed site-directed mutagenesis experiments on HBV core for mutations F23Y, L30F, T33Q, I105F and T109I and evaluated GLP-26 against these HBV core protein mutants. F23Y and L30F are resistance mutations for CAMs JNJ-6379 and BAY41-4109, while T33Q is a resistance mutation for SBA_R01, BAY41-4109 [47]. Thus, the selection of these five mutations covered the sensitivity and resistance for GLP-26 and novelty with respect to the resistance profile for other CAMs. GLS4, a well-known CAM, is resistant to T109I [47], so we tested GLS4 against T109I to validate our experiment.

**Figure 5:**
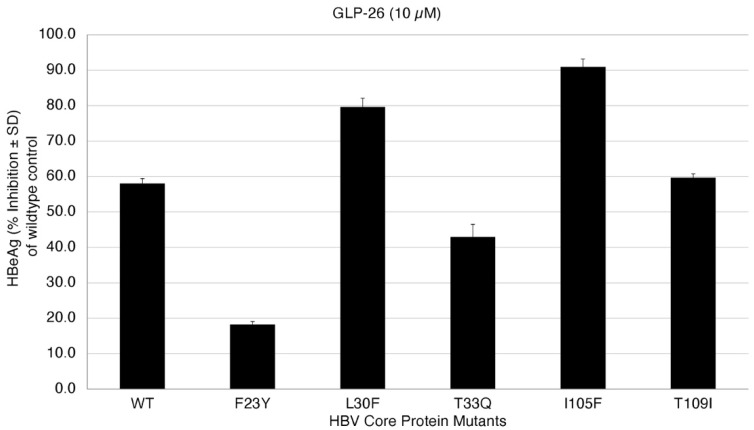
Inhibition of HBeAg secretion in HBV wild-type and core protein mutants at 10 µM GLP-26.

The effect of GLP-26 on HBeAg production (EC_50_) was measured (Figure 5) at 10 µM concentration. HBeAg production is a rapid and direct marker that is affected by capsid effector modulators in this transfection assay and is also largely cccDNA-dependent, and therefore can serve as a surrogate marker for cccDNA [49,50]. Interestingly, we were able to recapture the mutation profile predicted by our modeling approach (Table 3). GLP-26 was shown to be active against L30F, I105F and T109I mutants while T33Q and F23Y significantly decreased the GLP-26 effect on HBeAg production. We are currently evaluating the mutations which are predicted to be resistant to only GLP-26, but not to other CAMs.

**Figure 6:**
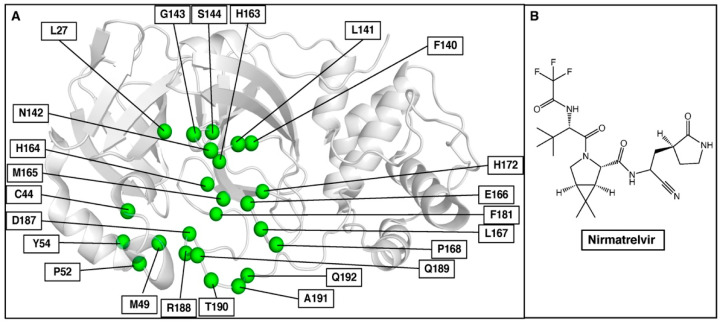
(**A**) Selected binding site residues in SARS-CoV-2 3CLpro to predict nirmatrelvir resistance mutations. (**B**) Chemical structure of nirmatrelvir.

Finally, we applied our prediction approach to SARS-CoV-2 3CLpro and the recently approved nirmatrelvir, which should be helpful in rapid assays to diagnose resistance and to select additional non-cross-resistant protease inhibitors. 3CLpro, one of the major therapeutic targets for anti-SARS-CoV-2 drugs, plays an important role in viral replication and cleaves polyprotein chains into non-structural proteins (NSPs). NSP peptide chains are the native substrates for 3CLpro. Thus, 3CLpro has 11 substrate peptides, and 3-D structures of six of them had been reported in Protein Data Bank (https://www.rcsb.org, accessed on 14 July 2022) complexed with 3CLpro when we started the work. The binding site residues of 3CLpro involved in this study are shown in Figure 6A.

Mutations were considered resistance mutations if they decreased nirmatrelvir binding affinity (ΔΔ*G* > 0 kcal/mol) but maintained or increased the binding affinity (ΔΔ*G* ≤ 0 kcal/mol) for at least three out of the six NSP substrates. The mutations identified using our approach are summarized in Table 4, and the list of prioritized resistance mutations is provided in supplementary materials. It is worth noting that these mutations were analyzed with a genomic database (https://www.gisaid.org (accessed on 14 July 2022)) to determine if any of these are known without drug treatment. Interestingly, Y54C was a mutant reported in March 2020 in Malaysia. Further, T190I has been reported 110 times from 15 different countries and represents 0.03% of the sequenced NSP5. As of now, there are no experimental or clinical resistance mutations reported in peer-reviewed articles on nirmatrelvir, but if our predictions are correct, these two naturally occurring variants could emerge with the use of nirmatrelvir and pose a severe threat to the population. We are currently evaluating the effect of the mutations on the binding of nirmatrelvir biochemically since our laboratory cannot perform gain-of-function studies with live coronaviruses.

Future work could involve estimating binding free energy change accurately for the predicted resistance mutations using the alchemical double-system/single-box method [51,52]. This would allow resistance mutations to be prioritized based on accurate binding free energies and help to identify drug resistance mutations which show the resistance due to large-scale conformational changes within or outside of the binding sites. Our computational approach could be explored further to study and predict double or multiple drug resistance mutations. It could also be used to explore drug resistance mutations in other viral protein targets (e.g., respiratory syncytial virus and Ebola virus).

## 3. Materials and Methods

### 3.1. Test System Selection and Preparation

HIV RT complexed with (-)-FTC-TP (PDB ID—6UJX), natural substrate and dCTP (PDB ID—6UIT) were selected to assess the approach to predict the resistance mutations. The crystal structure of GLP-26 with HBV core protein has not been resolved; therefore, our previously published modeled complex of GLP-26 with HBV core protein was selected for this study [44]. GLP-26 binds between two dimeric subunits and so tetramer HBV core protein (PDB ID—1QGT) was used. One additional system, SARS-CoV-2 3CLpro for nirmatrelvir (PDB ID—7RFS) was used to predict the resistance mutations. For the substrates, 3-D structures of SARS-CoV-2 3CLpro complexed with nsp4-nsp5 (PDB ID—7N89), nsp6-nsp7 (PDB ID—7DVX), nsp8-nsp9 (PDB ID—7MGR), nsp9-nsp10 (PDB ID—7DVY, nsp14-nsp15 (PDB ID—7DW6) and nsp15-nsp16 (PDB ID—7DW0) were used.

The PDB structures were prepared using Protein Preparation Wizard in Maestro (Schrödinger Release 2020-4; Schrödinger, New York, NY, USA). Missing residues and loops were added and minimized using Prime [53,54]. Crystallographic waters were deleted, and the hydrogen bonding network was optimized using Epik at neutral pH [55]. The final structures were minimized with heavy atom restraints using the OPLS3e force field. The minimization was terminated when the heavy-atom root mean square deviation reached 0.3 Å.

### 3.2. Residue Scanning

The binding site residues of drug/substrate were defined by Binding Site object in Maestro (Schrödinger Release 2020-4; Schrödinger). The change in binding affinities of the residues due to mutations was calculated using Residue Scanning module in BioLuminate (Schrödinger Release 2020-4; Schrödinger). Before residue scanning, a new chain ID was generated for drug/substrate/ligand molecule. In the residue scanning panel, all (allowed) mutations of interest residue were generated, and “Stability and Affinity” calculation was performed between drug/substrate/ligand chain and other binding partnered protein chains. During the calculations, for the refinement of the mutated residue, “Side-chain prediction with backbone sampling” option was selected with a cutoff of 0.0 Å. The residue scanning uses MM-GBSA refinement without any side-chain and backbone flexibility.

### 3.3. Prime MM-GBSA Calculations

The WT-drug/substrate and MUT-drug/substrate complexes which showed greater than 0 kcal/mol binding affinity change in residue scanning calculations were selected for Prime MM-GBSA estimation with side-chain flexibility. The protein complexes generated from residue scanning were split into ligand and protein structures which were selected for Prime MM-GBSA calculations. For the covalent systems, the covalent bond was removed for Prime MM-GBSA calculations. VSGB (variable-dielectric generalized Born) solvation model and OPLS3e force field were utilized during Prime MM-GBSA calculations. Side-chain flexibility was incorporated for the residues within 8 Å of the drug/substrate/ligand molecule by selecting a distance from ligand of 8 Å. For the sampling, the “minimize” option was selected. The changes in binding affinities, Δ*G*^MUT^ and Δ*G*^WT^, were calculated separately by Prime MM-GBSA, and the difference between them was used to calculate the change in the binding affinity due to mutations:

ΔΔ*G*_bind_ = Δ*G*^MUT^ − Δ*G*^WT^ (1)

### 3.4. Cell Lines

Wild-type HBV DNA was amplified and cloned as previously described (38, 39). Five HBV core mutants (F23Y, L30F, T33Q, I105F and T109I) were created by substituting nucleotides to change the codon as indicated below using the QuikChange II Site-Directed Mutagenesis Kit (Agilent, Santa Clara, CA, USA). Primers used for site-directed PCR mutagenesis are described in Table 5. The core genes of the mutants were sequenced bidirectionally by GENEWIX (South Plainfield, NJ, USA) to confirm the introduction of mutations.

HepNTCP-DL cells were maintained in Dulbecco’s modified minimal essential medium (DMEM) supplemented with 10% FBS and 0.1 mM non-essential amino acids (NEAA).

### 3.5. Compound Synthesis

GLP-26 and GLS4 were prepared in-house according to published procedures [45,56]. Both compounds had a purity of >95% as determined by 1H, 13C, 19F nuclear magnetic resonance (NMR) and high-pressure liquid chromatography (HPLC) analysis. Entecavir (ETV) was purchased from commercial vendors and confirmed at >95% purity using standard analytical methods such as mass spectrometry and NMR.

### 3.6. Transfection of Full-Length HBV DNA into HepNTCP-DL Cells

Full-length HBV DNA wild-type and core mutants were prepared for transfection as previously described [57]. HepNTCP-DL cells were seeded in either 96- or 24-well collagen-coated plates in DMEM supplemented with 10% FBS and 0.1 mM NEAA and maintained in a tissue culture incubator at 37 °C with 5% CO_2_. The cells were 90% confluent the next day, and the medium was changed to DMEM supplemented with 3% FBS and 0.1 mM NEAA. Transfection of HBV DNA was performed with Lipofectamine 3000 reagent (Invitrogen, Carlsbad, CA, USA) according to the manufacturer’s instructions. Twenty-four hours after transfection, the medium was replenished with drug-free medium or medium containing different concentrations of either GLP-26 or GSL4. Medium and cells (rinsed 3 times with ice-cold PBS) were harvested 3 days later. The efficiency of transfection was monitored by co-transfecting a β-galactosidase expression plasmid, pCMVβ (CLONTECH Laboratories Inc., Palo Alto, CA, USA). Assays for β-galactosidase in extracts of HuH-7 cells were performed as described [58]. Experiments were performed in triplicate.

### 3.7. Analysis of HBV HBeAg Production

Levels of HBeAg secreted in the culture medium were measured by using an HBsAg or HBeAg enzyme-linked immunosorbent assay (ELISA) kit (BioChain Institute Inc., Hayward, CA, USA), according to the manufacturer’s protocol. The concentration of compound that reduced levels of secreted HBeAg by 50% (EC_50_) was determined by linear regression.

**Table 5:** Primers used for site-directed PCR mutagenesis of HBV core.

Pair Name	Sequence Information
Core T109I Mutant c326t	5′-ctataactgtttctcttccaaaaatgagacaagaaatgtgaaaccac-3’
	5′-tgtgtttcacatttcttgtctcatttttggaagagaaacagttatag-3’
Core I105F Mutant a313t	5’-ccaaaagtgagacaagaaaagtgaaaccacaagagttgc-3’
	5’-gcaactcttgtggtttcacttttcttgtctcacttttgg-3’
Core T33Q Mutant	5’-atacagagctgaggcctgatctagaagatctcgtactgaaggaaaga-3’
a97c_c98a_c99g	5’-tctttccttcagtacgagatcttctagatcaggcctcagctctgtat-3’
Core L30F Mutant c88t	5’-gcggtatctagaaaatctcgtactgaaggaaagaagtc-3’
	5’-gacttctttccttcagtacgagattttctagataccgc-3’
Core F23Y Mutant t68a	5’-tctcgtactgaaggaaagtagtcagaaggcaaaaacg-3’
	5’-cgtttttgccttctgactactttccttcagtacgaga-3’

## 4. Conclusions

In this study, we assessed the ability of a computational approach containing residue scanning and prime MM-GBSA calculations to predict resistance mutations with an optimum balance between computational efficiency and accuracy. The approach successfully validated the prediction of the resistance mutations in HIV-RT for (-)-FTC-TP and can be used to predict the resistance mutations in HBV core protein for GLP26 and in SARS-CoV-2 3CLpro for nirmatrelvir. Three sensitivity mutations, L30F, I105F and T109I, and two resistance mutations, F23Y and T33Q, in HBV core protein for GLP26 were studied experimentally and validated our predictions. Hence, the approach demonstrated a strong correlation between prediction and experimental findings. Even though there are still areas for improvement such as accurately estimating binding affinity for the prioritization of resistance mutations, and predicting multiple drug resistance mutations, our approach could be used to develop drug treatment strategies for different antiviral agents, taking into account potential mutations that could arise and determining ways to minimize their selection in culture and in humans.

## Figures and Tables

**Table 1:** List of predicted resistance mutations in HIV-RT for (-)-FTC.

WT	Predicted Resistance Mutations
**M184**	**V, I**, A, R, N, D, C, Q, G, **L**, K, P, S, **T**, W, Y
I63	D, E
**K65**	**R**
**K66**	**I**, P, T, G
D113	E
Y115	A, R, N, D, C, Q, E, G, H, I, L, K, P, S, T, V
Q151	E, I, C, D, P
G152	D, E
Y183	D, E

Red bold—clinically reported (-)-FTC resistance mutations in HIV-RT.

**Table 2:** List of predicted resistance mutations in HBV core protein for GLP-26.

WT	Predicted Resistance Mutations
A132	**Y, W**, V, S
S106	**W, Y, F, L, R**, C
L140	**P, C, I, V, H, M**, Y
T128	**K, R, P, I, M**
L30	**W, H**, R
W102	**G, C, L, Y, F, S, R**
Y118	**Q, D, C, W, E, H, L, K, R**, F
P134	**L, H, S**
R133	**I, C, L**, T, H, M, G
W125	**G**
T33	**R, M**, Q, I
F110	**I**, L, M
P25	**L, Q**, R
F23	**M, L**, Y
I105	**M**, F, S, V, T
P138	**H**, A
P129	H, T, S, Q
F122	V, L, C
N136	R
V124	G, A
L37	P, Y, Q
R127	Q
A34	E

Bold—predicted resistance mutations in HBV core protein for GLP-26 with ΔΔ*G* > 3 kcal/mol.

**Table 3:** Experimental results for the selected mutations in HBV core protein for GLP-26.

HBV Core Protein Mutants	GLP26	Predictions for GLP26
WT	Sensitive	-
F23Y	**Resistant**	**Resistant**
L30F	Sensitive	Sensitive
T33Q	**Resistant**	**Resistant**
I105F	Sensitive	Sensitive
T109I	Sensitive	Sensitive

**Table 4:** List of predicted resistance mutations in SARS-CoV-2 3CLpro for nirmatrelvir.

WT	Predicted Resistance Mutations
S144	**Y, W**
H163	**Y**, Q
L167	**Q, P, F, R**, I, M, H
T190	**N, I, R**, K, A
R188	**H, K, N**, L
M49	**N, R**, K, F
P168	**S, Q**, R, A, H
Q192	**H, K, Y, L**, P
E166	**G, A**, K, V, N
F140	**S, I**, V, L
G143	**A, C**
Y54	**D, C, E**, Q, N, F, G, H, M, S, W, K
P52	**R**, S
H172	**Q**, N
H164	N, L, D, Q, K, T, E
Q189	H
A191	G, D
D187	G, Q, A
L141	Q, H
M165	L
F181	L, I, V, S, C
N142	I

Bold—predicted resistance mutations in SARS-CoV-2 3CLpro for nirmatrelvir with ΔΔ*G* > 3 kcal/mol.

## Data Availability

The data presented in this study are available on reasonable request.

## Supplementary Materials

The following supporting information can be downloaded at: https://www.mdpi.com/article/10.3390/molecules27175413/s1, Data S1: List of mutations with binding free energy change.
